# The Effects of Synthetically Modified Natural Compounds on ABC Transporters

**DOI:** 10.3390/pharmaceutics10030127

**Published:** 2018-08-09

**Authors:** Daniel Dantzic, Pawan Noel, Fabrice Merien, Dong-Xu Liu, Jun Lu, Haiyong Han, Mark J. McKeage, Yan Li

**Affiliations:** 1 School of Science, Auckland University of Technology, Auckland 1010, New Zealand; daniel.dantzic@aut.ac.nz (D.D.); fabrice.merien@aut.ac.nz (F.M.); dong-xu.liu@aut.ac.nz (D.-X.L.); jun.lu@aut.ac.nz (J.L.); 2 Translational Genomics Research Institute, Phoenix, AZ 85004, USA; pnoel@tgen.org (P.N.); hhan@tgen.org (H.H.); 3 AUT-Roche Diagnostics Laboratory, Auckland 1010, New Zealand; 4 School of Interprofessional Health Studies, Auckland University of Technology, Auckland 0627, New Zealand; 5 College of Life Sciences and Oceanography, Shenzhen University, Shenzhen 518061, China; 6 College of Food Engineering and Nutritional Science, Shaanxi Normal University, Xi’an 710062, China; 7 Department of Pharmacology and Clinical Pharmacology, University of Auckland, Auckland 1023, New Zealand; m.mckeage@auckland.ac.nz; 8 Auckland Cancer Society Research Centre, University of Auckland, Auckland 1023, New Zealand

**Keywords:** ABC transporter, drug disposition, multidrug resistance, P-glycoprotein (P-gp), breast cancer resistant protein (BCRP), multidrug resistance-associated proteins (MRPs)

## Abstract

Multidrug resistance (MDR) is a major hurdle which must be overcome to effectively treat cancer. ATP-binding cassette transporters (ABC transporters) play pivotal roles in drug absorption and disposition, and overexpression of ABC transporters has been shown to attenuate cellular/tissue drug accumulation and thus increase MDR across a variety of cancers. Overcoming MDR is one desired approach to improving the survival rate of patients. To date, a number of modulators have been identified which block the function and/or decrease the expression of ABC transporters, thereby restoring the efficacy of a range of anticancer drugs. However, clinical MDR reversal agents have thus far proven ineffective and/or toxic. The need for new, effective, well-tolerated and nontoxic compounds has led to the development of natural compounds and their derivatives to ameliorate MDR. This review evaluates whether synthetically modifying natural compounds is a viable strategy to generate potent, nontoxic, ABC transporter inhibitors which may potentially reverse MDR.

## 1. Introduction

**Figure 1:**
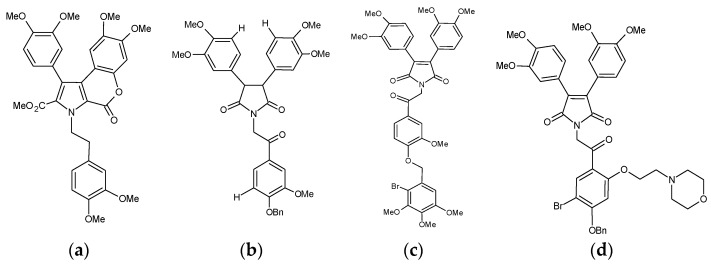
Ningalin B compounds which act as potent P-gp inhibitors, from references 45, 49, 50 and 51. (**a**) N3 [49]. (**b**) Compound 35. 1-(2-(4-(benzyloxy)-3-methoxyphenyl)-2-oxoethyl)-3,4-bis(3,4-dimethoxyphenyl)-1H-pyrrole-2,5-dione 1-(2-(2-bromo-5-methoxy-4-((3,4,5-trimethoxybenzyl)oxy) phenyl)-2-oxoethyl)-3,4-bis(3,4-dimethoxyphenyl)-1H-pyrrole-2,5-dione [50]. (**c**) Compound 23. 1-(2-(2-bromo-5-methoxy-4-((3,4,5-trimethoxybenzyl)oxy) phenyl)-2-oxoethyl)-3,4-bis(3,4-dimethoxyphenyl)-1H-pyrrole-2,5-dione [45]. (**d**) Compound 12. 1-(2-(4-(Benzyloxy)-5-bromo-2-(2-morpholinoethoxy)-phenyl)-2-oxoethyl)-3,4-bis(3,4-dimethoxy-phenyl)-1H-pyrrole2,5-dione [51].

Multidrug resistance (MDR) is the process by which cells become resistant to multiple unrelated drugs [1,2]. One of the main mechanisms by which cancer cells become resistant is up-regulation of various ABC transporters such as P-glycoprotein (P-gp), Breast Cancer Resistant Protein (BCRP) and Multidrug Resistant Protein 1 (MRP1) which efficiently remove the drug from the cell, thus causing the drug to lose its effect [3]. There are 48 human ABC transporters, all with the ability to utilize ATP as an energy source to transport substrates across cell membranes [3,4]. ABC transporters are made up of two components—the transmembrane domain (TMD) which creates the passageway for substrates to pass through membranes and the nucleotide-binding domain (NBD), where ATP is hydrolyzed [5]. This provides the energy required for the conformational change of the TMD from the closed to the open conformation, which is essential for the effective transport of small molecules across the membrane [5]. Significant cellular accumulation of anticancer drugs is attenuated by select ABC transporters because of their ability to efflux anticancer substrates out of cancer cells independent of concentration gradients (Figure 1) [6]. MDR can also arise from mutations in drug targets such as p53, increased DNA repair mechanisms, inhibition of apoptotic signaling, high glutathione levels, and increased rates of drug metabolism [7,8]. The ability to re-sensitize cancer cells to chemotherapeutics has been extensively studied as it believes that when the limitation of resistance is removed, the efficacy of cancer treatment will greatly improve [8,9].

The activity of ABC transporters has become an essential consideration when designing drugs, as ABC transporters greatly affect pharmacokinetics, toxicity and efficacy of drugs [10]. Absorption, distribution, metabolism, excretion and toxicity are the properties that most dictate the success of a new drug, all of which can be affected by the expression of ABC transporters [11], which ABC transporters are present in key organs such as the intestines, liver, kidneys, blood brain barrier and blood placental barrier [12]. ABC transporter has also impacted the development of anticancer drugs from natural bioactives. The natural compound camptothecin (CPT) and its derivatives show a variety of antitumor activities [13] but high BCRP expression caused a 400–1000-fold increase in resistance towards camptothecin derivatives [14]. CPT is a substrate of P-gp ad MRP2 and its erratic oral bioavailability has been attributed to the intestinal and biliary excretion mediated by P-gp and MRP2 [15]. Resveratrol is another natural compound that is associated with low bioavailability [16]. Despite of its beneficial effects towards different diseases such as cardiovascular disease, cancer and neuerodegenerative diseases, the low bioavailability of reseveratrol can be mainly attributed to the efficient elimination by intestinal BCRP and phase II metabolism [16]. The natural compound apigenin has been highlighted for its chemopreventative affects [17]. There has also been a large amount of in vivo data showing the anticancer effects of apigenin against multidrug resistant tumours, leukemia models and osteosarcoma xenografts [18,19,20]. Saeed et al., investigated the relationship between apigenin and ABC transporter expression [21]. Apigenin accumulation remained unaffected by P-gp expression, while, apigenin treatments showed inhibition of both P-gp and BCRP [21]. Genistein much like resveratrol exhibits low bioavailability in clinic which could be related to its broad interactions with BCRP, P-gp and MRP2 [22,23,24]. All of these natural, highly bioactive, poorly bioavailable compounds may also rely on ABC transporter inhibitors or synthetic modification to improve their activity in clinic.

The first generation of P-gp modulators verapamil and cyclosporine A were approved for other diseases and then readily tested for their MDR reversal efficacy in clinic [25,26]. However these synthetic compounds showed little MDR reversal, low potency and poor pharmacokinetics while exhibiting high toxicity and serious off target effects such as heart block and in one case congestive heart failure in the case of verapamil [25]. The second generation of P-gp modulators valspodar and dexverapamil derivatives of first generation P-gp inhibitors were designed for increased specificity [26]. In vitro results showed higher potency and specificity however in clinic the second generation modulators showed little MDR reversal effects and various side effects such as neutropenia and high toxicity [25], because they also inhibit CYP450-mediated anti-cancer drug metabolism which lead togreatly increased systemic exposure of cytotoxics and thus enhanced toxicity [25]. Third generation inhibitors such as tariquidar and zosuquidar were developed with improved potency and minimized CYP450 inhibitory activity [25]. They were more potent with less pharmacokinetic interactions, however, increased toxicity of chemotherapy regimens was still seen with limited clinical benefit [26].

Designing new, potent MDR reversal agents which are naturally occurring compounds or structurally related to naturally occurring compounds is currently of considerable interest [27]. In clinic, curcumin a poorly bioavailable natural compound is well-tolerated, shows limited pharmacokinetic interactions and has significantly improved disease stability in colorectal cancer [28]. Synthetically modifying well-tolerated natural compounds has the potential to create derivatives with increased specificity, potency, and improved bioavailability [27,29]. Much like the development of P-gp inhibitors it may take the use of structure activity relationship studies and continual synthetic improvements of previously created compounds to create compounds that can be successful in clinic. The majority of papers in this review use an ABC transporter overexpressing cell line to evaluate the specificity of the inhibitors being tested and permitting a comparison with inhibitors and ABC transporter expression. In some cases, researchers have examined how their inhibitors react with multiple ABC transporters, which lets researchers further evaluate the specificity of these compounds across different ABC transporters. This is especially important when considering the broad overlap in inhibition and substrate specificity of some of the ABC transporters [30]. The purpose of this review is to evaluate the success of synthetically modified natural compounds in reversing MDR in cancer.

Database searches were conducted until 18 June, 2018 (articles in English only). This included Pubmed, Scopus and MEDLINE. Sets of key words and their combination search terms were used, including “derivatives”, “analogue”, “analog”, “natural compound”, “phytochemical”, “flavonoid”, “phytonutrient”, “plant chemical”, “transport”, “ABC transporter”, “ATP-Binding Cassette”, “MRP”, and “multidrug resistance”. An article was only considered if it contained a derivative of a natural compound AND natural compound AND ABC transporter. The titles and abstracts were initially evaluated and the full texts were further reviewed. A total of 266 studies were identified, and 59 were ultimately selected for discussion in this review.

## 2. ABCB1/P-gp

In 1973, it was observed that daunorubicin, vincristine, and vinblastine all appeared to be actively transported from resistant Ehrlich ascite tumor cells [31]. In 1976, P-gp a surface glycoprotein was isolated from a colchine-resistant cell line, P-gp was given its name due to the effects that it exhibited on the cell membrane permeation [32]. It was found that the amount P-gp present correlated with the resistance observed against colchine [32]. The MDR1/ABCB1 gene, discovered in 1986, encodes the P-gp protein [33]. P-gp is a 170 kDa protein with 1280 residues in a single polypeptide chain [32,34]. P-gp is expressed at various crucial barriers within the body, including the intestinal wall, the blood–brain barrier (BBB), the placental barrier, in addition to being expressed in key waste organs, including the liver and kidneys [3,35,36]. The expression of P-gp at the apical side of the intestinal wall and the BBB suggests its role in limiting the entry of drugs into the bloodstream and the central nervous system, by pumping drugs either back into the gut lumen or back into the bloodstream, which under normal circumstances would act as a protective measure against toxins [3,37]. However, in the context of drug development and cancer treatment ABC transporters can play a large role in drug pharmacokinetics and therapeutic efficacy [26,37]. It was for this reason that the American Food and Drug Administration (FDA, Silver Spring, MD, USA) created strict guidelines on how drug and ABC transporter interactions should be evaluated during drug development [26]. P-gp transports a variety of anticancer agents against concentration gradients, including vinblastine, paclitaxel, doxorubicin, erlotinib, colchicine, doxorubicin and teniposide [34,38]. Given the widespread use of these chemotherapeutics, inhibition of P-gp may transform relatively poorly performing cytotoxic drugs into exceptional ones.

The expression of P-gp is found at high levels in the adrenal glands and the kidneys, moderate expression in the lung, liver, lower jejunum, colon and, rectum and low expression in a variety of other tissues [39]. In clinic high expression of P-gp is only found in a few tumor types such as renal cancer and pheochromocytoma [39,40]. The majority of tumor samples show much less expression when compared with resistant cell lines [39,40]. A recent paper demonstrated that the level of expression of P-gp correlated with the amount of resistance exhibited [41]. At high P-gp concentrations such as seen in overexpression model resistance towards nilotinib was minimal but significant [41]. At a low P-gp expression levels nilotinib resistance is lost [41]. The MDR reversal capabilities of nilotinib and imanitib both showed successful reversal of MDR when the expression of P-gp was at low to moderate levels, however, at higher P-gp expression levels nilotinib MDR reversal required higher concentrations which initiated off target affects [41]. The antiproliferative, anticancer agent ABT-263 and ABT-199 is another compound that is able to reverse MDR but only when BCRP expression is low [42]. This research suggests a potential clinical relevance of using low, moderate and highly efficient ABC transporter inhibitors depending on the expression level of ABC transporters within the tumors. It also suggests that much of the research and designs for MDR modulators has not been wasted.

### 2.1. Ningalin B

The P-gp inhibitory activity of natural marine compounds including Ningalin B and terpenoids was comprehensively summarized in the review article by Long et al. [43]. Ningalin B is a natural marine product isolated from the ascidian (sea squirt) family of the genus *Didemnum* [44,45,46]. In the earliest ningalin study a number of synthetic intermediates (e.g., *O*-methyl ningalin B, compounds 10, 11, 13 and 14) were synthesised from the ningalin isolate [44]. While ningalin B showed moderate cytotoxicity in L1210 and HCT116 cell lines with IC_50_ values of 10 and 12 µM, respectively [44]. The synthetic analogue *O*-methyl ningalin B was 5-fold and 2.5-fold less cytotoxic than ningalin B, respectively, in L1210 and HCT116 cell lines [44]. P-gp overexpressing HCT116/VM46 cells showed increased resistance to doxorubicin and vinblastine [44]. Ningalin compounds 10, 11, 13 and 14 at 1 µM potently sensitized HCT116/VM46 cells towards doxorubicin and vinblastine [44]. Compound 14 (1 µM) increased the cytotoxicity of vinblastine to the point where the HCT116/VM46 resistant cells became more sensitive to vinblastine than the HCT116 wild type cells [44]. Unmodified ningalin B was, however, unable to reverse MDR in HCT116/VM46 [47]. This suggests that synthetic modification of natural products can generate more potent, moderately toxic, P-gp specific, MDR reversal agents. Subsequent studies of other ningalin B analogues demonstrated low toxicity and potent MDR reversibility towards doxorubicin and vinblastine in HCT116/VM46 resistant cells [47,48]. Ningalin B analogues 3 and 4 (1 µM) caused a complete reversal in MDR of vinblastine and a 50% reduction in resistance towards doxorubicin in a P-gp dependent manner [47]. Further modification of compound 3 led to the generation of ningalin B derivatives 19, 20 and 21 that exhibited complete MDR reversal towards doxorubicin and vinblastine without the toxicity associated with compound 3 [47,48].

Six additional ningalin analogues (N1-N6) reported by Chou and colleagues showed a wide range of cytotoxicity responses, with IC_50_ values ranging from 13 µM (N3) to 150 µM (N4) in vinblastine-sensitive cells (CCRF-CEM), and 18 µM (N5) to 250 µM (N2) in vinblastine-resistant cells (CCRF-CEM/VBL100) [49]. All the ningalin analogues except N5 showed lower toxicity in vinblastine-resistant cells (CCRF-CEM/VBL100) compared with vinblastine-sensitive cells (CCRF-CEM), suggesting that N1-N4 and N6 are P-gp substrates [49]. An increase in sensitivity ranging from 210-fold (N1) to 6.2 × 10^6^-fold (N3) was observed for vinblastine and paclitaxel by co-incubation of CCRF-CEM/VBL_100_ cells with 10 µM ningalin analogues [49]. In particular, N3 (Figure 1) sensitized vinblastine-resistant cells (CCRF-CEM/VBL_100_) towards vinblastine and paclitaxel to a level greater than that observed for vinblastine-sensitive cells (CCRF-CEM) [49]. A combination study revealed that combining ningalins and doxorubicin resulted in a reduction in the IC_50_ of both compounds. The strong synergism between ningalins and doxorubicin in doxorubicin resistance cells was confirmed using the chou-talalay method [49]. In vivo, whilst paclitaxel treatment slowed tumor progression, the addition of N3 also resulted in tumor shrinkage, and, in one case, complete elimination of the tumor [49]. Several in vitro P-gp function assays demonstrated that ningalins compete for [^3^H]azidopine binding to P-gp, increase the cellular accumulation of VBL or paclitaxel, and inhibit drug efflux from the tumor cells. These results indicate that the synergistic antitumor activity between ningalins and chemotherapeutic drugs could be due to the inhibition of P-gp by ningalins.

The P-gp overexpressed breast cancer cell line MDA435/LCC6MDR was used to investigate the most recently designed ningalin analogues by direct comparison with wild type MDA435/LCC6 (Table 1) [45,50,51,52]. Doxorubicin accumulation assays showed that compounds 6, 25, 12, 23, 35 and 37 caused a 3.0, 2.1, 2.6, 2.4, 2.2 and 2.3-fold increase in doxorubicin accumulation in resistant cell lines, respectively (Table 2) [45,50,52]. The potency of compounds based on the doxorubicin accumulation from the most to the least potent were as follows: 37 > 35 > 23 > 12 > 6 > 25 [45,50,52]. These compounds were also nontoxic towards normal human fibroblast L929 cells LCC6 [45,50,51,52]. The combination of compounds 6 and 23 showed a greater response towards doxorubicin accumulation than when used individually [52]. Compounds 35 and 37 did show some selectivity towards P-gp in that they not inhibit MRP1 transport and only moderately affected BCRP transport [50].

Doxorubicin and rhodamine 123 are relatively specific substrates of P-gp. Quantifying the accumulation levels of specific P-gp substrates within the cell correlates with the activity of the P-gp transporter [45,50,52]. Compounds 12 (Figure 1) (2 µM) and 23 (2 µM) increased rhodamine 123 accumulation by 3.9 and 4.8-fold, respectively [45,51]. Compound 23 showed an EC_50_ of 78 nM which was 4.7-fold less than the typical P-gp inhibitor verapamil [45]. The synthetic ningalin compounds 6, 25, 35, 37, 12 and 23 proved nontoxic and more potent than verapamil with respect to inhibiting P-gp transport. Furthermore, compounds 12, 23, 35 and 37 were more effective in reversing MDR towards vinblastine than verapamil, and re-sensitized resistant cells towards other drugs to a greater extent than non-resistant cells [44,45,50,51,52].

Structure-activity analysis (SAR) studies were carried out to investigate whether increasing the number of methoxy groups on ring B of ningalin B, changing the polarity, extending the linking chain, and substituting ring C with other functional groups would improve the potency and cytotoxicity of permethyl ningalin B analogues [50]. A significant improvement in potency and cytotoxicity were observed when the benzyloxy group was attached at the C ring in compounds 35 and 37 [50]. It was found that the number of methoxy substituents played a role in determining P-gp modulation, although the extent to which this occurs was not precisely established [50]. There was also evidence that more polar N substituents at the ring C decreased the P-gp activity [50]. SAR analysis of other ningalin compounds synthesized by Yang et al. including compound 23, showed that addition of a para-trimethoxybenzyloxy, an ortho-bromo and a meta-methoxy group at ring C were important pharmacophores for P-gp modulation in ningalin compounds [45].

### 2.2. Tetrandrine

The phytochemical tetrandrine (TET) is a bisbenzylisoquinoline, isolated from Stephania tetrandra roots which has been used in China since the 1960s for treatment of silicosis lesions [58,59]. TET was shown by Fu et al. to increase the cellular accumulation of a P-gp substrate Fura-2 in a concentration-dependent manner [58]. Furthermore, in the presence of TET at 0.625, 1.25 and 2.5 µM, doxorubicin-resistant cells (MCF-7/ADR) were re-sensitized to doxorubicin by 5.4, 11.8 and 20.4-fold, respectively [58]. Mice bearing subcutaneous MCF-7/ADR tumor xenografts were also re-sensitized to doxorubicin by concurrent treatment with TET without a significant increase in toxicity [58].

**Figure 2:**
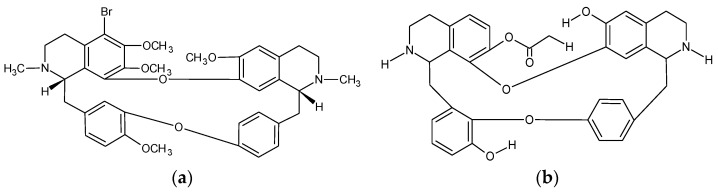
Structures of 5-bromotetrandrine and W6. (**a**) Bromotetrandrine (11S,31S)-35-bromo-16,36,37,54-tetramethoxy-12,32-dimethyl-11,12,13,14,31,32,33,34-octahydro-2,6-dioxa-1(7,1),3(8,1)-diisoquinolina-5(1,3),7(1,4)-dibenzenacyclooctaphane [53]; (**b**) W6 [54].

A brominated analogue of TET, bromotetrandrine (bromoTET, Figure 2), was reported to have a significant sensitization effect on vincristine, doxorubicin, paclitaxel, docetaxel and epirubicin in P-gp overexpressing KBv200 (multidrug resistant) cells but not in the parental KB cells [53]. BromoTET also caused a significant increase in doxorubicin accumulation in a concentration dependent manner in KBv200 cells [53]. BromoTET alone resulted in a reduction of tumor growth at 7.5 mg/kg and 10 mg/kg by 14.8 and 23.5%, respectively, in KBv200 xenograft nude mice [53]. Co-administration of 10 mg/kg Bromo-TET and 2 mg/kg epirubicin significantly enhanced the antitumor activity of epirubicin without increasing the toxicity [53]. BromoTET also increased the accumulation of doxorubicin within the KBv200 xenograft tissue while leaving the P-gp mRNA and protein expression unaffected, indicating that TET derivatives specifically inhibit P-gp transport function [53]. However bromoTET did show some limitations, with high toxicity in cancerous KB and KBv200 cell lines (IC_50_ values of 5.14 and 6.17 µM, respectively) [53].

H1, a synthetic derivative of TET, also showed P-gp inhibition evidenced by increased doxorubicin and rhodamine 123 accumulation (3.7 and 29.7-fold increase) in KBv200 cells when treated with 0.5 µM H1 [59]. H1 caused a complete reversal of resistance to doxorubicin and a partial reversal of resistance to vincristine and paclitaxel in KBv200 cells [59]. KB-sensitive cells remained unaffected by H1 in cellular accumulation experiments [59]. Cisplatin (a well-established MRP2 substrate) toxicity remained unaffected by incubation with H1, since cisplatin is not transported by P-gp. These results suggest that H1 is a relatively specific P-gp modulator [59]. How these compounds interact with P-gp was probed by their effects on ATPase activity, which is an indicator of NBD binding and transport activation [59]. Compounds interacting with an ABC transporter can stimulate or inhibit its ATPase activity, which is measured by the amount of inorganic phosphate generated by ATP hydrolysis [59]. H1 inhibits the ATPase activity of P-gp but is not a P-gp substrate because of the lack of ATPase stimulation effects [59]. H1 also decreases the protein expression of P-gp in a concentration dependent manner but the mRNA levels of ABCB1 (MDR1) are unaffected [59]. Further analysis revealed that P-gp expression was reduced by ubiquitination which is modulated by the MEK-ERK signalling pathway [59]. Similar to bromoTET, H1 was also inherently cytotoxic, with IC_50_ values ranging from 2-10 µM for the cell lines investigated [59].

W6 (Figure 2), was also shown to be a highly potent MDR reversal agent [54]. In the drug resistant KBv200 and MCF-7/DOX cells, 1 µM W6 increased the accumulation of doxorubicin by 4 and 5.3-fold, respectively [54]. W6 also reversed MDR, lowering the IC_50_ values of vincristine, doxorubicin and paclitaxel by 27.8, 29.2 and 1050-fold, respectively, in resistant KBv200 cells, and 64.5, 30.3 and 99.3, respectively, in MCF-7/DOX cells [54]. Like H1, W6 inhibited ATPase in a dose-dependent manner and decreased the protein expression of P-gp while the mRNA levels were unaltered [54]. These results suggest that W6 inhibits P-gp in a noncompetitive manner, which was confirmed by photo labelling experiments [54]. Knockdown of ERK1/2 has previously been shown to inhibit the expression of P-gp, and W6 significantly decreased the expression of ERK1/2 in a time dependent manner [54]. H1 and W6 appear to show some commonality in the mode of action and the extent of cytotoxicity [54].

### 2.3. Terpenes

Celastraceae sesquiterpenes are isolated from the Celastraceae plant family which for centuries has been used to treat a variety of diseases [60]. Sesquiterpenes exhibit a wide range of biological activities, suggesting that they can interact with multiple proteins, including P-gp [60,61]. The major contributor to the variety of biological activities exhibited are the terpenoid compounds which are structurally diverse secondary metabolites [61]. Celastraceae is also made up of non-terpenoid secondary metabolites such as quercetin, another well natural product that exhibited MDR reversal activities [61]. Twenty eight dihydro-β-agarofuran sesquiterpenes isolated from various celastraceae plants showed reduced cytotoxicity in the drug-sensitive NIH-3T3 cell line, while showing increased cytotoxicity in the P-gp expressing cells, suggesting some collateral sensitivity [49,60]. Collateral sensitivity is a phenomenon by which cells resistant to one drug become hyposensitized to another [62]. Machu4 and Mama12 sesquiterpenes showed the most potent inhibition of P-gp-mediated daunorubicin efflux, with IC_50_ values of 0.24 and 0.33 µM, respectively [60]. Machu4, Mama5, and Mama12 reversed vinblastine resistance at 1 µM and were 5 to 9-fold more potent than verapamil [60]. Compounds which can substitute [^3^H] Azidopine from the binding pocket are likely to interact directly with the P-gp binding site [60]. The most potent daunorubicin efflux inhibitor, Machu4, did not decrease [^3^H] Azidopine photolabelling, unlike the second most potent compound, Mama12 [60]. These compounds were also highly specific P-gp inhibitors which showed no MRP1, MRP2 and BCRP modulation [60]. The demonstrated ability of sesquiterpenes to selectively modulate P-gp activity in vitro by both traditional and unique binding interactions with P-gp makes these compounds an interesting target for continued investigation.

Callies and co-workers prepared 58 dihydro-B-agarofuran sesquiterpene derivatives that showed a greater inhibitory activity against P-gp-mediated efflux of daunorubicin compared with verapamil [63]. Six of the analogues (6, 24, 50, 57, 58 and 59) showed higher inhibitory potency than the lead dihydro-B-agarofuran compound [63]. Eighteen of the compounds showed vinblastine re-sensitization, five compounds showed greater MDR reversal activity than verapamil, and compound 48 completely reversed MDR at 3 µM [63]. Dihydro-B-agarofuran sesquiterpenes showed weak binding affinities and similar cytotoxic levels across control and MDR cell lines, suggesting that sesquiterpenes inhibit P-gp in a non-competitive manner. This is a useful property for MDR reversal agents [63].

**Figure 3:**
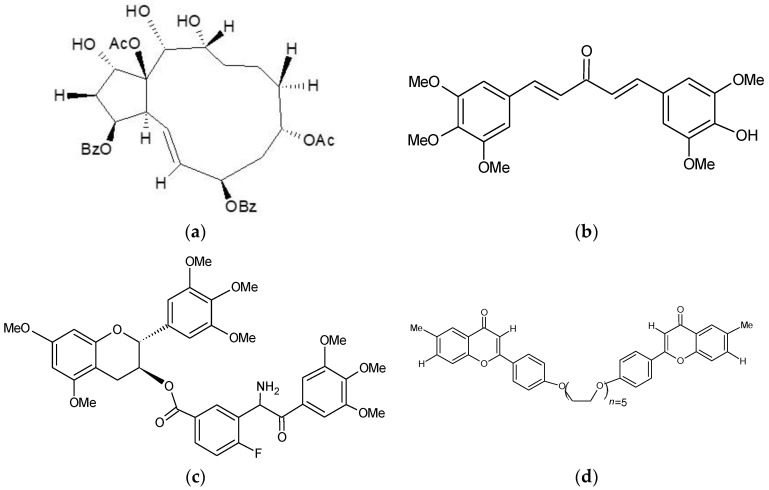
Other Potent MDR reversal agents. (**a**) Compound 26. (1S,2S,3S,4S,7R,9R,13R,14R,15S)-9,15-Fiacetoxy-3,7-dibenzoyloxy-1,13,14-trihydroxyjatropha-5E-ene [55]. (**b**) GO-Y078. (1E,4E)-1-(4-hydroxy-3,5-dimethoxyphenyl)-5-(3,4,5-trimethoxyphenyl)penta-1,4-dien-3-one [29]. (**c**) Compound 51. (2R,3S)-5,7-Dimethoxy-2-(3,4,5-trimethoxyphenyl)chroman-3-yl. 3-(3,4,5-trimethoxybenzamido)-4-fluorobenzoate [56]. (**d**) Compound 4e. 1,16-Bis[40-((6-methyl)-4H-chromen-4-on-2-yl)phenyl]-1,4,7,10, 13,16-hexaoxahexadecane [56].

Jatrophane diterpenoids are isolated from the Euphorbia family of plants and have shown potent and specific inhibition of P-gp [55,64]. Compound 6 was twice as potent as the typical P-gp inhibitor cyclosporine A [64]. Further in vitro and in vivo evaluation of the jatrophane diterpenoid synthetic derivatives revealed that several compounds (12, 26, 29 and 35) inhibited rhodamine 123 efflux more efficiently than verapamil at 1 µM [55]. These unique compounds showed no cytotoxicity in resistant (up to 150 µM) and sensitive cell lines (up to 100 µM), while displaying strong MDR reversal for doxorubicin [55]. Compound 26 (Figure 3) was the most potent jatrophane MDR reversal agent, reducing doxorubicin IC_50_ values by 61 and 36-fold in HepG2/ADR and MCF-7/ADR resistant cells, respectively [55]. In vivo, a combination of compound 26 and doxorubicin showed a significant reduction in the final tumour volume and an increase in overall survival in a HepG2/ADR xenograft model [55]. Other diterpenes also demonstrated MDR reversal properties such as macrocyclic diterpene derivatives, which were structural modifications of the lead compound jolkinol D. These compounds were more cytotoxic than jolkinol D [65]. Five of the compounds showed reduced rhodamine 123 efflux at lower concentrations compared with higher concentrations [65]. All except jolkinol D showed synergism with doxorubicin incubation in L5178/MDR1 cells [65]. Myrsinol diterpenes exhibited mild toxicity, and compound J196-9-4 stimulated ATPase and inhibited rhodamine 123 efflux but only at high concentrations, suggesting that J196-9-4 was a competitive P-gp inhibitor [66].

### 2.4. Other Notable Synthetic P-gp Inhibitors

The broad activity and substrate specificity of P-gp can be further exhibited by the broad structural diversity of potential P-gp inhibitors. Methylated epigallocatechin, gallocatechin, and dihydromyricetin derivatives (compounds 23, 35, and 36) were not cytotoxic (>100 µM) for LCC6, LCC6MDR (a P-gp overexpressing breast cancer cell line) and L929 fibroblast cell lines [56]. The nontoxic compounds 23, 35 and 36 inhibited doxorubicin efflux while at 3 µM compounds 23 and 36 completely reversed MDR [56]. Methylated epigallocatechin and gallocatechin derivatives reversed MDR towards paclitaxel, vinblastine, vincristine, and doxorubicin in the nanomolar range (EC_50_ between 102 to 280 nM) which were more potent than verapamil [56]. Intracellular accumulation of compounds 23 and 51 (Figure 3) in both MDR and parental cells were comparable, suggesting that they are not P-gp substrates [56]. Structure-activity analysis revealed some important P-gp inhibition pharmacophores including methoxy, allyloxy, or acetylamino substitutions at ring D and rigid linkers of oxycarbonylvinyl and oxycarbonylphenylcarbamoyl with optimal link lengths ranging from 7.75 to 13.37 Å between the D and C3 rings. 6,7-Dimethoxy-2-phenethyl-1,2,3,4-tetrahydroisoquinoline derivatives showed strong P-gp/calcien-AM accumulation inhibition [67]. Compounds 4 and 10 potently inhibited calcein-AM efflux, with EC_50_ values of 0.3 and 0.33 µM, respectively [67]. Sing et al. endeavoured to model the small intestinal wall in vitro by using a Caco-2 cell monolayer system [67]. The bi-directional transport of compounds 4 and 10 across the Caco-2 monolayers were not in an active manner, suggesting they are not P-gp substrates [67]. This is also consistent with the fact that they neither stimulate P-gp ATPase activities. Much like Dihydro-B-agarofuran sesquiterpenes, tetrahydroisoquinoline derivatives also inhibited P-gp in a non-competitive manner.

Several coumarin derivatives were reported to be more potent in blocking P-gp mediatedrhodamine 123 efflux than verapamil [68]. They were also highly cytotoxic across the sensitive and resistant cell lines studied [68]. However, compounds 1a, 1e, 1f, 1g, and 1h all showed collateral sensitivity with more than a two-fold increase in cytotoxicity against the doxorubicin-resistant Lovo/Dox cells in comparison to sensitive cells [68]. Cell cycle analysis revealed that the coumarin derivatives induce significant cell cycle changes [68]. Compound 1b induced significant G0/G1 arrest, 1c caused S phase arrest and 1e at lower concentrations caused the G0/G1 arrest and at high concentrations caused G2/M block [68]. This suggests that coumarin derivatives may target other sites that are more essential for cell survival.

Tiamulin, a nontoxic semi-synthetic antibiotic, was capable of reversing MDR against colchine, doxorubicin, and vinblastine [69]. daunorubicn accumulation was increased after treatment with 2 µM tiamulin by 5.3-, 2.3- and 4.0-fold in P-gp expressing AS30-D/COL5, CEM/VLB3.6, and P388/ADR25 cells, respectively [69]. In vivo, overall animal survival was increased by 29% with tiamulin treatment [69]. Reserpine and yohimbine analogues showed high vinblastine MDR reversal. Unfortunately, these analogues also showed high cytotoxicity [38]. Certain flavone derivatives, especially the aurone derivatives, significantly increased paclitaxel accumulation in MDR cells and bound strongly to P-gp [70]. Quercetagetin, a methoxyflavone, was the most potent [^3^H] vincristine efflux inhibitor, while all methoxyflavones tested in the study showed significant inhibition of [^3^H] vincristine at 0.2 µM [70]. Hydrocinchonine, cinchonine, and quinidine reversed MDR against paclitaxel and docetaxel in MDR cells by activation of PARP [35]. P-gp specific modulation by hydrocinchonine, cinchonine, and quinidine was confirmed by increased rhodamine 123 accumulation [71].

Quercetin has been shown to modulate multiple ABC transporters including P-gp, BCRP and MRP1 [34,72]. Active quercetin derivatives showed low toxicity (IC_50_ > 100 µM) while displaying high P-gp modulating activity [72]. Compound 17 at 1 µM increased paclitaxel sensitivity by 11.3-fold [72]. Bivalent quinine inhibitors Q(6′,6′) and Q(6′,4′) showed calcein AM efflux inhibition [73]. Honokiol, Magnolol and 4-*O*-Methylhonokiol compounds are nontoxic and improve daunorubicin resistance, however, honokiol was the only compound capable of weakly inhibiting calcein-AM efflux [74]. Alpha-tocopherol metabolites and gamma-tocotrienol weakly inhibited rhodamine 123 efflux, while gammaT3 was the only compound to significantly inhibit rhodamine 123 transport [75]. Typically, inhibitors reduce P-gp expression but gammaT3 increased P-gp protein expression while downregulating its transport [75].

## 3. ABCG2/BCRP

Breast cancer resistance protein (BCRP) is also a member of the ABC transporter family and is responsible for the transport of a variety of anticancer drugs including but not limited to mitoxantrone, topotecan, irinotecan, doxorubicin, SN-38, flavopiridol, and methotrexate [76,77,78,79]. BCRP has significant overlap with P-gp and MRP1 in substrate specificity, tissue distribution and expression [30,80]. Much like P-gp, the inhibition of BCRP reverses MDR, which could lead to improved therapeutic outcomes [76,79]. However, Ko143, a third generation synthetic inhibitor of BCRP showed poor results in vivo [26]. Ko143 showed highly potent inhibition of BCRP in vitro however in vivo Ko143 exhibited a short plasma half-life of approximately 1 h [81]. Thereafter, natural compounds including flavonoid scaffolds, aurones, marine products, quinazoline and chalcone moieties and protoflavones have all been modified to investigate BCRP reversibility [77,78,82,83,84,85].

Aurones are a family of flavonoids previously shown to reverse MDR towards mitoxantrone in ABCG2-overexpressing cells [83]. Mitoxantrone, pheophorbide A and Hoechst 33342 are fluorescent BCRP substrates which can be detected by flow cytometry, allowing direct quantification of BCRP transport activity [78,83,84]. Aurone analogues A-2, A-3, I-2, I-3, F-2 and F-3 exhibited considerable cellular accumulation of mitoxantrone, up to levels comparable to fumtremogin C (FTC), a well-defined BCRP inhibitor [83]. Four methoxylated aurone compounds at 50 nM significantly reduced the IC_50_ of mitoxantrone [83]. Mitoxantrone was combined with 0.5 µM of a series of chalcone (A-2, A-3, I-2, C-2) compounds and the F-2 aurone compound resulted in complete MDR reversal in resistant cells [83]. Moreover, I-2 sensitized cells resistant towards mitoxantrone to a higher extent than that observed in control cells [83]. ATPase assays revealed that the potent analogues A-2, A-3, I-2, F-2 were strong ATPase stimulators [83]. However, they are not BCRP substrates, since there are not different growth inhibitory effects for these compounds on ABCG2-overexpressing (MDA-MB-231/R) and parental (MDA-MB-231/V) breast cancer cells [83]. Compounds A-2, I-2 and F-2 showed a weak association with the [I]IAAP/BCRP binding site, whereas A-3 completely inhibited [I]IAAP photolabelling [83]. These compounds all show some unique binding and inhibitory characteristics with BCRP. In vivo the combination of A-2 and mitoxantrone significantly improved overall survival when compared with mitoxantrone treatment alone [83].

Quinazoline and chalcone compounds both show BCRP inhibitory activity at high nanomolar to low micromolar concentrations [84]. By combining the two moieties to generate new synthetic derivatives, Kraege et al. endeavoured to obtain more potent BCRP inhibitors [84]. Compound 35 inhibited pheophorbide A efflux in a BCRP overexpressing cell line with an IC_50_ of 0.19 µM, which is well below cytotoxic levels [84]. Compound 35 also showed a 10-fold larger therapeutic ratio than that of Ko143, a potent but highly cytotoxic BCRP inhibitor [84,86]. Compound 35 revealed no selective difference in toxicity for MDR and sensitive cells, suggesting that compound 35 was not a substrate of BCRP as it remained unaffected by increased BCRP expression/transport [84]. However, compound 35 still potently reversed SN-38 resistance at 0.01 µM and 0.1 µM [84]. Compound 35 showed high specificity towards BCRP in comparison to other ABC transporters [84]. Compound 35 potently inhibited BCRP, slightly modulated P-gp and did not affect MRP1 transport [84]. Most of the quinazoline compounds showed relatively high toxicity, with IC_50_ values of less than 10 µM across the sensitive and resistant cell lines [78]. The therapeutic ratio of drugs is dependent upon the toxicity shown in comparison to the intended inhibitory activity [78]. Quinazoline derivatives 21, 54 and 60 exhibited improved therapeutic ratios in comparison to Ko143 even when considering these compounds’ high cytotoxicity [78]. The large therapeutic window can mainly be attributed to the potent inhibition of Hoechst 33342 efflux by compounds 21, 54 and 60, with IC_50_ values of 55.6, 44.2 and 47.5 nM, respectively [78]. Compounds 54 and 60 also potently sensitized MDR cells towards SN-38 (EC_50_ values of 12.7 nM and 15.6 nM respectively) and mitoxantrone (EC_50_ values 7.4 nM and 9.7 nM respectively) [78]. ATPase analysis revealed that these potent inhibitors followed a bell-shaped curve [78].

Curcumin is a phytochemical derived from the readily available spice turmeric which has been extensively investigated for its biological activity [87,88,89]. Observed activities include MDR reversal, antioxidant, anti-inflammatory, and anti-cancer activities [89,90,91]. However, the oral bioavailability of curcumin is very limited due to it is poor intestinal absorption, rapid metabolism into curcumin conjugates and a short half-life [92,93,94]. Curcumin also showed the ability to inhibit a broad spectrum of ABC transporters including P-gp, MRP1, MRP5 and BCRP transport across the respective overexpressing cell lines [27]. However, curcumin analogues A12, B11 and C10 showed increased potency and specificity than curcumin but they only inhibited a single ABC transporter [27]. A12 (IC_50_: 1.2 µM) and B11 (IC_50_: 5.2 µM) were more potent than curcumin (IC50: 32 uM) in BCRP modulation [27]. C10 (IC_50_: 2.8 µM) only inhibited P-gp exhibiting increased specificity and potency compared with curcumin (50.5 uM) [27]. Interestingly A13 was a multi-specific inhibitor of BCRP, MRP1 and MRP5, which was shown to possess superior potency compared with curcumin [27].

Murakami et al. screened 24 nontoxic synthetic curcumin analogues with increased bioavailability to evaluate their efficacy in BCRP modulation [29]. The curcumin analogues GO-Y078 (Figure 3), GO-Y0168, GO-Y0172 and GO-Y030 potently inhibited mitoxantrone transport, with IC_50_ values of 0.51, 0.31, 0.25 and 0.37 µM, respectively, while curcumin exhibited an IC_50_ of 0.62 µM [29]. The desire GO-Y030 (1 µM) exhibited complete MDR reversal and GO-Y078 (1 µM) greatly enhanced SN-38 sensitivity in MDR cells while GO-Y0168 and GO-Y0172 were inactive [29]. GO-Y078, GO-Y0168, GO-Y0172 and GO-Y030 modulated ATPase activity and [I]IAAP binding at nanomolar concentrations, exhibiting high binding affinity the BCRP binding site [29]. This suggests that GO-Y030 and GO-Y078 show competitive inhibition of BCRP by competing for the substrate binding pocket of BCRP [29]. However, GO-Y0168 and GO-Y0172 also showed strong binding and mitoxantrone transport inhibition, yet only GO-Y078 and GO-Y0168 were able to reverse SN-38 resistance [29]. This suggests that curcumoid-mediated SN-38 MDR reversal is not entirely dependent on BCRP binding [29]. In vivo GO-Y078 exhibited a 1.4-fold increase in survival that could be attributed to its increased solubility and bioavailability when compared with curcumin [95]. However this in vivo activity could be attributed to the specificity of these potent P-gp inhibitors which also show some weak BCRP transporter inhibition [29]. Curcumin is a typical example of synthetic modification successfully improving the bioavailability, potency, and specificity of the lead bioactive compound [95].

## 4. ABCC1/MRP1

MRP1 transports a wide variety of xenobiotics and metabolites essential for cancer treatment and has been associated with poor prognosis, much like P-gp [96,97]. However unlike P-gp MRP1 is localized to the basolateral membrane of mucosal cells, therefore, under normal conditions MRP1 is responsible for transporting substrates into the bloodstream [98]. The complexity and cross-reactivity of these transport mechanisms can be well exhibited by MRP1. Glutathione at high concentrations react with xenobiotics creating glutathione conjugates thereby limiting there xenobiotics activity [99]. It has been suggested that MRP1 further enhances resistance by transporting these xenobiotic glutathione conjugates [99]. The nontoxic bivalent apigenin homodimer compound 4e (Figure 3, IC_50_ > 100 µM) potently inhibited MRP1-dependent doxorubicin efflux and completely reversed MDR against both doxorubicin and etoposide at 0.5 µM (Table 1) [56,57]. SAR showed removal of OH groups at the C-5 and C-7 positions of the A-ring from apigenin dimers and methyl substitutions at the C-6 or C-7 or fluorine substitution at the C-7 position of the Å ring of the dimer showed the highest increase in MRP1-modulating activity [57]. The high binding affinity of 4e within the MRP1 substrate/doxorubicin binding site showed that 4e inhibits transport by competitive inhibition [57].

The investigation of BCPCF (2′,7′-bis-(3-carboxy-propyl)-5-(and-6)-carboxyfluorescein) accumulation, which is an MRP1 specific substrate, resulted in relatively low potencies with IC_50_ values of 13, 16, 16, 7, 5 and 7 µM for Licoisofalvone B, IFG10, IFG12, morin, silybin and indomethacin, respectively [100]. Several nontoxic lignan derivatives showed even less potency upon BCPCF accumulation, with IC_50_ values ranging from 50 to 125 µM [100]. The naturally occurring compound oleanolic acid which exhibits anticancer properties was synthetically modified to create the DIOXOL and HIMOXOL derivatives [101]. DIOXOL and HIMOXOL showed an 8-fold increase in calcein-AM fluorescence in comparison to MK-571, a potent MRP1 inhibitor [101]. While DIOXOL and HIMOXOL both inhibited MRP1-mediated transport in MDR cells, they also showed strong anti-proliferative activities such as DNA fragmentation, increased Bax expression and decreased BCL-2 expression [101]. Further development of MRP1 inhibitors could help elucidate the mechanism for MRP1-associated MDR in cancer cells [100].

## 5. Multi-Specific ABC Transporter Inhibitors

It has been shown that some tumour types actually express multiple ABC transporters [26]. In vivo, it was shown that knocking out both BCRP and P-gp increased drug accumulation 43-fold compared with the knockout of only BCRP or P-gp [102]. As previously mentioned P-gp, BRCP and MRP1 all have some overlap in the substrates and co-localize at the same tissue [80,103]. This suggests a potent multi-specific ABC transporter inhibitor could simultaneously block multiple efflux pathways and potentially increase the efficacy of anti-cancer drugs. Daunorubicin and epirubicin while vinblastine, mitoxantrone, topotecan and etoposide are transported by either P-gp and MRP1 or P-gp and BCRP [103,104].

Cyclosporine A is an example of a multi-specific ABC transporter inhibitor in that it can inhibit MRP1, BCRP and P-gp [105]. Cyclosporine A showed an increase in clinic in disease-free survival while PSC-833, a derivative of cyclosporine A which only inhibited P-gp did not show such improvements [105]. Quercetin derivatives such as compound 17 was not only the most potent P-gp inhibitor but also inhibited BCRP by 7.3-fold [72]. Curcumin derivatives have been shown to inhibit the widest range of ABC transporters, including P-gp, BCRP, MRP1 and MRP5 [27]. Twenty three heterocyclic cyclohexanone curcumoids were tested for P-gp, BCRP, MRP1 and MRP5 inhibition [27]. In cellular accumulation assays of transporter specific substrates, compound C10 showed strong and specific P-gp inhibition (IC_50_: 2.8 µM) in resistant cell lines while A13 showed varying activities with respect to BCRP, MRP1 and MRP5, with IC_50_ values of 4.3, 11.9 and 11.7 µM, respectively [27]. Substrate inhibition also translated to an increase in drug sensitivity, with compound C10 causing MDR reversal of paclitaxel resistance and A12 and A13 resulting in MDR reversal of mitoxantrone resistance [27]. 6,7-Dimethoxy-2-phenethyl-1,2,3,4-tetrahydroisoquinoline derivatives 8, 9 and 12 were capable of modulating both calcein-AM and Hoescht 33342 efflux in their respective P-gp and BCRP expressing cell lines [27,67].

Quinazoline and chalcone derivative 24 was designed to inhibit BCRP, yet it showed equipotent inhibition against BCRP and P-gp, with IC_50_ values of 0.6 and 0.48 µM, respectively [84]. Compounds 19 and 27 both showed stronger inhibition of P-gp and then BCRP and MRP1 transport inhibition [84]. Methylated epigallocatechin, gallocatechin and dihydromyricetin derivatives were also found to inhibit multiple ABC transporters [56]. While compounds 23 and 35 completely reversed P-gp dependent doxorubicin resistance, they also showed strong MDR reversal towards BCRP [56]. Moreover, derivatives 50 and 51 showed activity against all three ABC transporters MRP1, BCRP and P-gp [56]. High specificity to a single target is usually a highly regarded characteristic for drug design, however further investigation into multi-specific ABC transport inhibitors would appear essential in this case as inhibition of multiple ABC transporters may be required to substantially reduce MDR in the clinic [26,104].

## 6. Future Directions

The development of P-gp inhibitors showed gradual improvement in clinic with the use of synthetic modification such as the creation of valspodar and dexverapamil derivatives of verapamil and cyclosporine A [26]. Natural compounds which possess a vast range of biological activities can be harnessed and synthetically modified to overcome MDR. Synthetic derivatives with low intrinsic toxicity can be used to improve MDR reversal, pharmacokinetcal properties beyond that of their parental compounds [47,53,55,73]. The compounds discussed in this review show potent efflux inhibition and, in some cases, complete MDR reversal at low, nontoxic concentrations, which warrants further investigation in vivo [82].

The clinical data presented on MDR reversal agents has been greatly hindered by the clinical study design. The earliest clinical trials used AML as a model, yet only 13% of patients expressed P-gp, which is understandable considering there was no effective way to evaluate P-gp expression [26]. Therefore, screening of patients for the target ABC transporter expression is required to ensure that the expression levels are present and at a level that can actually respond to inhibition [106]. However this can also be misleading as mRNA and protein expression levels do not always correlate with transport function [107]. The fast and efficient detection of ABC transporters functional activity would be a key step in better evaluating the clinical relevance of MDR reversal agents [26]. The ability of ^99m^Tc-Sestamibi to effectively detect not only P-gp expression but transport function was demonstrated in pancreatic cancer cell [107].

Protoflavones exhibit collateral sensitivity in MDR cells as they exhibited higher cytotoxicity against MDR cell lines MCF-7/dox and KB-V1 cells that overexpress P-gp and BCRP, respectively [85]. With the exception of protoapigenone the 51 remaining protoflavones showed no response to the overexpression of BCRP/P-gp [85]. The bypassing of ABC transport by designing anticancer drugs that aren’t transported by ABC transporters could also be a viable strategy to overcome MDR resistance [85]. The evaluation of less potent ABC inhibitors in models where ABC transporter expression are closer to the clinical levels should be investigated further.

The substrate and inhibition overlap of MRP1, BCRP and P-gp could suggest the need for multi-specific ABC transport inhibitors [30]. A recent paper by Stefan K. et al., revealed that this strategy was feasible [108]. Nine-deazapurines multi-specific ABC transport inhibitors have been made [108]. The most promising compound, compound 55 was able to restore sensitivity towards P-gp, BCRP and MRP1 at submicromolar concentrations with a therapeutic ratio greater than 9 [108]. A potential limitation of for multi-specific ABC transport inhibitors is that they modulate individual ABC transporters at different potency which could make it difficult to establish an effective dosage in patients [105]. Nevertheless, evaluation of multi-specific ABC transport inhibitors may prove beneficial in clinic. In fact the only compound to show slight improvement in clinic cyclosporine A is a multi-specific ABC transport inhibitor [105]. It is important to note that the activity of other ABC transporters such as MRP2, 3, 4 and, 5 have also exhibited the ability to confer MDR [109,110,111,112]. The substrate specificity and expression level of these “known unknown” ABC transporters vary significantly across cancers [5,30,103]. The inhibition of these transporters could create more clinically relevant strategies to reverse MDR.

## Figures and Tables

**Table 1:** Comparison of the potent synthetic modulators in vitro.

Reference	Compound	Target	Cytotoxicity (µM)			
Ting-Chao Chou [49]	N3	P-gp	CCRF-CEM: 13	CCRF-CEM/VBL1000: 100		
Bin [50]	Compound 35	P-gp	L292: >100	MDA435/LCC6: >100	MDA435/LCC6MDR: >100	
Yang [45]	Compound 23	P-gp	L292: >100	MDA435/LCC6: >100	MDA435/LCC6MDR: >100	
Wang [51]	Compound 12	P-gp	L292: >100	MDA435/LCC6: >100	MDA435/LCC6MDR: >100	
Chen [53]	5 Bromo-tetrandrine	P-gp	KB: 5.14	KBv200: 6.17		
Sun [54]	W6	P-gp	KB: ~2	KBv200: ~4	MCF-7: ~4.5	MCF-7/DOX: ~5
Zhu [55]	Compound 26	P-gp	HepG2/ADR: >150	MCF-7/ADR: >150		
Kraege [29]	GO-Y078	BCRP	K562/BCRP: 0.31			
Wong [56]	Compound 51	MRP1, P-gp and BCRP	L292: >100	LCC6: >100	LCC6MDR: >100	
Wong [56]	4e	MRP1	L292: >100	LCC6: >100	LCC6MDR: >100	

**Table 2:** Effects of the potent synthetic modulators on cellular accumulation and cytotoxicity of substrates of ABC transporters.

Compound	Target	Cellular Accumulation in MDR Cells (Relative-Fold)		MDR Reversal in MDR Cells (Relative-Fold)				
N3 [49]	P-gp			Vinblastine: 440				
Compound 35 [50]	P-gp	Doxorubicin 1 µM: 2.2		Paclitaxel: 42.7				
Compound 23 [45]	P-gp	Doxorubicin 1 µM: 2.4		Paclitaxel: 48.0				
Compound 12 [51]	P-gp	Doxorubicin 1 µM: 2.6		Paclitaxel: 39.8				
5 Bromo-tetrandrine [53]	P-gp	Doxorubicin 1.5 µM: ~1.2		Doxorubicin: 15.6	Vincristine: 109.4	Paclitaxel: 78.4	Docetaxel: 57.8	Epirubicin: 25.1
W6 [54]	P-gp	Doxorubicin KBv200 1 µM: 4	Doxorubicin MCF-7/DOX 1 µM: 5.3	KBv200 Doxorubicin: 27.8 MCF-7/DOX Doxorubicin: 30.3	KBv200 Vincristine: 29.2 MCF-7/DOX Vincristine: 64.5	KBv200 Paclitaxel:1049.6 MCF-7/DOX Paclitaxel: 99.3		
compound 26 [55]	P-gp	Rhodamine 123 HepG2/ADR 2 µM: 2.74		HepG2/ADR 100 nM Doxorubicin: 71	MCF-7/ADR 200 nM Doxorubicin: 36			
GO-Y078 [29]	BCRP	Pheophorbide A 1 µM: >3		SN-38: 1.18				
compound 51 [56]	MRP1, P-gp and BCRP	Doxorubicin 2008/MRP1 1 µM: 2.6	Doxorubicin HEK293/R2 (BCRP expressing) 1 µM: 10.4	Paclitaxel: 31.4				
4e [56]	MRP1	Doxorubicin 2008/MRP1 1 µM: 8.9						
4e [57]	MRP1	Doxorubicin 3 μM: 2.1		0.5 µM Doxorubicin: 13.7	0.5 µM Etoposide: 10.2			
